# Enhancing pathological complete response prediction in breast cancer: the role of dynamic characterization of DCE-MRI and its association with tumor heterogeneity

**DOI:** 10.1186/s13058-024-01836-3

**Published:** 2024-05-14

**Authors:** Xinyu Zhang, Xinzhi Teng, Jiang Zhang, Qingpei Lai, Jing Cai

**Affiliations:** 1 Department of Health Technology and Informatics, The Hong Kong Polytechnic University, Hong Kong, China; 2 The Hong Kong Polytechnic University Shenzhen Research Institute, Shenzhen, China

**Keywords:** Breast cancer, Radiomics, DCE-MRI, Treatment response prediction

## Abstract

**Background:**

Early prediction of pathological complete response (pCR) is important for deciding appropriate treatment strategies for patients. In this study, we aimed to quantify the dynamic characteristics of dynamic contrast-enhanced magnetic resonance images (DCE-MRI) and investigate its value to improve pCR prediction as well as its association with tumor heterogeneity in breast cancer patients.

**Methods:**

The DCE-MRI, clinicopathologic record, and full transcriptomic data of 785 breast cancer patients receiving neoadjuvant chemotherapy were retrospectively included from a public dataset. Dynamic features of DCE-MRI were computed from extracted phase-varying radiomic feature series using 22 CAnonical Time-sereis CHaracteristics. Dynamic model and radiomic model were developed by logistic regression using dynamic features and traditional radiomic features respectively. Various combined models with clinical factors were also developed to find the optimal combination and the significance of each components was evaluated. All the models were evaluated in independent test set in terms of area under receiver operating characteristic curve (AUC). To explore the potential underlying biological mechanisms, radiogenomic analysis was implemented on patient subgroups stratified by dynamic model to identify differentially expressed genes (DEGs) and enriched pathways.

**Results:**

A 10-feature dynamic model and a 4-feature radiomic model were developed (AUC = 0.688, 95%CI: 0.635–0.741 and AUC = 0.650, 95%CI: 0.595–0.705) and tested (AUC = 0.686, 95%CI: 0.594–0.778 and AUC = 0.626, 95%CI: 0.529–0.722), with the dynamic model showing slightly higher AUC (train *p* = 0.181, test *p* = 0.222). The combined model of clinical, radiomic, and dynamic achieved the highest AUC in pCR prediction (train: 0.769, 95%CI: 0.722–0.816 and test: 0.762, 95%CI: 0.679–0.845). Compared with clinical-radiomic combined model (train AUC = 0.716, 95%CI: 0.665–0.767 and test AUC = 0.695, 95%CI: 0.656–0.714), adding the dynamic component brought significant improvement in model performance (train *p* < 0.001 and test *p* = 0.005). Radiogenomic analysis identified 297 DEGs, including CXCL9, CCL18, and HLA-DPB1 which are known to be associated with breast cancer prognosis or angiogenesis. Gene set enrichment analysis further revealed enrichment of gene ontology terms and pathways related to immune system.

**Conclusion:**

Dynamic characteristics of DCE-MRI were quantified and used to develop dynamic model for improving pCR prediction in breast cancer patients. The dynamic model was associated with tumor heterogeniety in prognostic-related gene expression and immune-related pathways.

**Supplementary Information:**

The online version contains supplementary material available at 10.1186/s13058-024-01836-3.

## Introduction

Breast cancer is one of the most common malignant in women. In 2020, there were around 2.3 million women newly diagnosed with and over 600,000 women died of breast cancer worldwide [1]. Recently, neoadjuvant chemotherapy (NAC) has become increasingly used in breast cancer systemic treatment. NAC was initially used in inoperable breast cancer to enable surgical resection, and expanded to other types of breast cancer for increasing the chance of breast conservation owing to its remarkable efficacy [2]. Current NAC treatment schemes are determined by hormone receptor (HR) status and human epidermal growth factor receptor 2 (HER2) status as recommended by American Society of Clinical Oncology (ASCO) [3]. Pathological complete response (pCR), defined as no residual disease in breast and axillary region after NAC, is a validated prognostic factor to assess treatment response and associated with long-term outcome [4]. However, only 10-50% patients achieved pCR, varying according to their receptor subtypes [5]. Moreover, the assessment of pCR status is performed at surgery after completion of NAC, prior to which non-responders have suffered from the toxicity and side effects caused by NAC. Therefore, it is essential to identify patients who are likely to achieve pCR before NAC to avoid unnecessary complications and maximize potential benefits.

Dynamic contrast-enhanced MRI (DCE-MRI) is the clinical routine for breast cancer assessment. It has high sensitivity in diagnosis and treatment monitoring [6, 7]. Through acquisition of sequential images before, during, and after the administration of contrast agent, DCE-MRI provides valuable information about tissue perfusion and contrast agent enhancement dynamics associated with tumor angiogenesis [8]. Radiomics extracts high-dimentional image features that are imperceptible to human eyes to non-invasively quantify tumor characteristics [9]. Radiomic analysis of breast DCE-MRI has been used for pCR prediction in many previous studies, most of which only used radiomic features from one or several phases while ignoring the dynamic information [10, 11]. Recently, attempts have been made to leverage the dynamic information embedded in DCE-MRI for pCR prediction by combining radiomic features extracted from different DCE-MRI phases. For instance, Peng et al. calculated delta-features between two different phases for pCR prediction [12]; Li et al. employed simple statistical patterns of radiomic features extracted from different phases for pCR prediction and achieved better performance compared to single-phase features, demonstrating the value of multi-phase information [13]. In BMMR2 challenge, radiomic features from kinetic maps, such as peak enhancement maps and signal enhancement ratio maps, were used to predict pCR [14]. However, the entire time series of radiomic features has not been fully explored and may contain additional information for tumor characterization. On the other hand, feature-based representation of time series data like 22 CAnonical Time-series CHaracteristics (Catch22) can capture the dynamic properties of time series data and was used in various tasks [15, 16]. Accordingly, there developed an assumption that the dynamics of radiomic feature series extracted by Catch22 can characterize the dynamic information in DCE-MRI and improve pCR prediction of breast cancer patients.

**Fig. 1:**
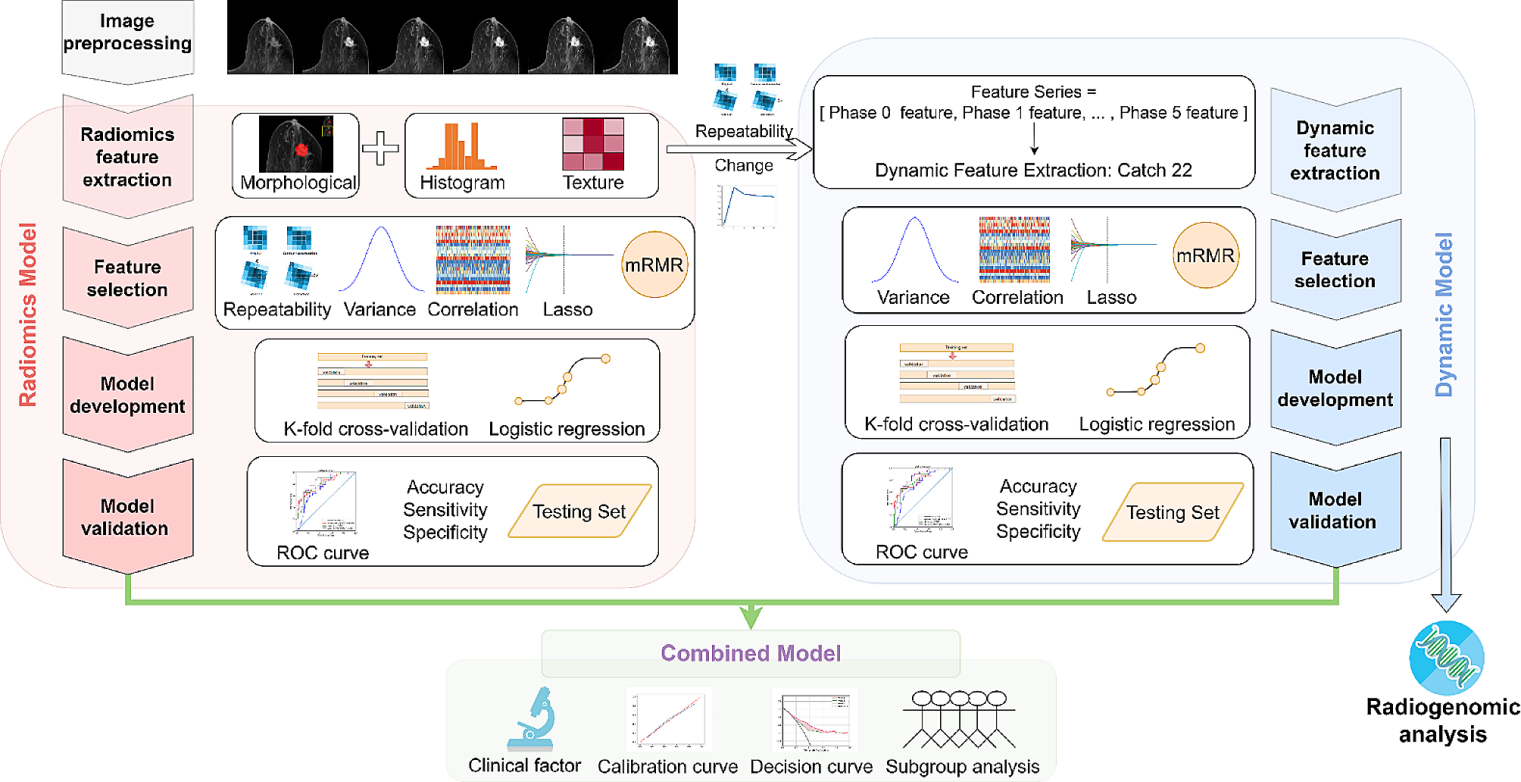
Workflow of the study. Firstly, the collected DCE-MR images were preprocessed by normalization and discretization. Radiomic features were extracted from multiple phases of DCE-MRI, while dynamic features were extracted from radiomic feature series. Feature selection, model development, and model validation were then conducted separately for radiomic model and dynamic model. Subsequently, combined models were developed by integrating radiomic, dynamic, and clinical information and their performance were evaluated. In addition, radiogenomic analysis was performed on dynamic model to investigate potential biological mechanisms

In this study, we aimed to systematically extract dynamic properties of radiomic feature series from DCE-MRI to improve treatment response prediction of breast cancer patients. To achieve this, a large number of dynamic features were extracted by Catch22 from DCE-MRI feature series, and a dynamic model was then built for pCR prediction. Various combinations of dynamic models and existing radiomic and clinical models were developed to find the optimal one as the final model. In addition, radiogenomic analysis of binarized dynamic model predictions was conducted to explore its association with tumor heterogeneity and biological process. Figure 1 shows the overall workflow of this study.

## Materials and methods

### Patient data

A total of 985 stage II/III locally advanced breast cancer patients enrolled in the multi-center I-SPY2 trial (clinical trial number: NCT01042379) during 2010 to 2016 were collected from the publicly available dataset on The Cancer Image Archive [17–19]. Institutional review board approval was waived due to the use of public data. The detailed descriptions of I-SPY2 trial have been reported by previous paper [20]. All the patients underwent MR examination and percutaneous biopsy before receiving NAC. After the completion of NAC, patients underwent surgical resection to assess residual disease. The exclusion criteria included: (1) incomplete image or clinicopathologic data; (2) deviations from the prescribed scanning protocol; (3) insufficient image quality.

### Clinicopathologic data

Clinicopathologic data including HR, HER2, MammaPrint status (MP), pCR, and other patient characteristics was provided by the dataset. HR and HER2 were deternmined by immunohistochemical (IHC) staining or fluorescence in-situ hybridization (FISH) of tissues obtained during pre-treatment biopsy. HR was determined as positive when ≥ 5% tumor staining for ER and/or PgR was seen. HER2 was determined as positive by IHC 3 + or FISH overexpression [21]. The surrogate of treatment response pCR was defined as no residual disease in breast and axillary lymph nodes after NAC and obtained by post-treatment surgery [22].

### Imaging data and tumor segmentation

The scanning process of DCE-MRI can be found on TCIA website [17, 18]. DCE-MRI scanning protocol details are provided in Supplementary Material Table S1. The pre-contrast phase and five post-contrast phases were used for radiomic feature extraction and subsequent analysis.

The region of interest was segmented by functional tumor volume (FTV) included in the dataset. The calculation of FTV involved background filtering, estimating signal enhancement ratio, and applying a peak enhancement threshold in a manual-defined 3D bounding box [23].

### Image preprocessing and feature extraction

Image normalization was performed across different DCE-MRI phases of the same patient to preserve the dynamic information. All the images were isotropically resampled to 1*1*1mm^3^, and discretized by a fixed bin width of 5. More details of image preprocessing can be found in Supplementary Material Figure S1. Radiomic features were extracted from each phase of DCE-MRI using PyRadiomics package version 3.0.1 following the standardization and definitions in the image biomarker standadization initiative [24, 25]. The extracted features included morphological features (*n* = 14), first-order features (*n* = 17), and texture features (*n* = 79). The repeatability of radiomic features was evaluated by perturbation which involved random translation, rotation, and contour randomization of original masks [26–28]. Features with high-repeatabliity (intraclass correlation coefficient, ICC ≥ 0.9 [29]) were retained for better model repeatability. The fluctuation of radiomic features was measured by performing a single-sample t test on the variations between features from different phases. Radiomic feature series were constructed by concatenating the high-repeatable and phase-varying first-order and texture features. Dynamic features were extracted from radiomic feature series using the 22 CAnonical Time-series Characteristics (catch22) feature set, specifically designed for capturing the dynamic properties of time series data, such as distributions and outliers, linear and non-linear autocorrelation, and so on [30].

### Model development and evaluation

The development of radiomic model and dynamic model followed the same process including feature selection and model building. The features with low variances were removed first to retain those providing more information. Then, features with significant correlations with pCR were identified by MannWhitney U test, where a p value smaller than 0.05 was defined as significant. LASSO was subsequently used to select the independently discriminative features. Finally, features were ranked by minimum redundancy and maximum relevance (mRMR) algorithm considering the relevance to pCR and redundancy at the same time [31]. Clinical factors that are commonly used in clinical decision making and have significant associations with pCR were identified and used in developing clinical model. Combined models were constructed using logistic regression with clinical factors, prediction score of the dynamic model, and prediction score of radiomic model as variables. Different combination strategies were adopted, including combining two of the three variables respectively, as well as the combination of all three together. The independence of the components in the combined models were examined by their coefficients and p values. All the models were developed using Logistic Regression with 10-fold cross-validation in training set and tested in independent testing set.

The pCR prediction performance of the candidate models was assessed by various metrics, including area under receiver operating characteristic curve (AUC), accuracy, sensitivity, and specificity. AUCs were calculated by continuous prediction (the probability) and the other metrics were calculated by binary prediction (pCR or non-pCR) dichotomized by Youden index. An AUC typically ranges from 0 to 1 while AUC equal to one means a perfect descrimination ability. The optimal model was determined by the highest internal validation AUC in the training set. Heatmap was employed to visualize the relationships between different models and their association with clinical factors. SHapley Additive Explanations (SHAP), a method to interpret and explain the output of machine learning models, was employed to evaluate the importance of each component in the model with the highest AUC [32]. In our case, where the model output is the probability of achieving pCR, the SHAP values for each parameter ranges from − 1 to 1 and a larger absolute value means a higher importance for model output. Calibration curves and Brier scores were used to further evaluate the alignment between model-predicted probabilities and actual probabilities. Brier score measures the accuracy of probabilistic predictions and takes the value from 0 to 1, for which 0 means a perfect prediction. Decision curve analysis was performed to evaluate the clinical benefit obtained by the optimal model [33]. Besides, to further demonstrate the generalizability of the optimal model, its association with pCR in various pre-defined molecular subtypes, namely HR + HER2-, HR + HER2+, HR-HER2-, and HR-HER2+, and patients receiving different treatments were evaluated.

### Radiogenomic analysis

To examine whether the dynamic model can reflect tumor heterogeneity and its association with gene expression, we collected paired total mRNA expression data from National Center for Biotechnology Information (NCBI) [34]. Patients were divided into DYN + and DYN- groups according to the binary prediction of dynamic model. Student t test was performed to identify differentially expressed genes (DEGs) between the two groups. An absolute log-2 fold change larger than 0.25 and a p value smaller than 0.05 were used as cutoff. Enriched Gene Ontology (GO) terms and Kyoto Encyclopedia of Genes and Genomes (KEGG) pathways were identified by gene set enrichment analysis (GSEA) of DEGs [35–39]. A p value smaller than 0.05 and false discovery rate (FDR) smaller than 0.25 were considered statistically significant.

### Statistical analysis and software

For statistical analysis, Chi-Square test or Fisher’s exact test was used for categorical variables and MannWhitney U test was used for continuous variables. A two-tailed p-value smaller than 0.05 was considered statistically significant. The 95% CIs for AUCs were calculated according to DeLong’s methods [40]. DeLong test was used to compare the AUCs of two independent models and likelihood ratio test was used to compare the model fit of nested models to demonstrate the improvements conferred by the additional factors in complex models. The statistical analysis was carried out on R4.2.2 [41] and Python3.7 [42]. Logistic regression was carried out by package scikit-learn 1.0.2 [43]. Radiogenomic analysis was conducted using packages scanpy 1.9.3 [44] and gseapy 1.1.0 [45].

## Results

### Patient characteristics

**Fig. 2:**
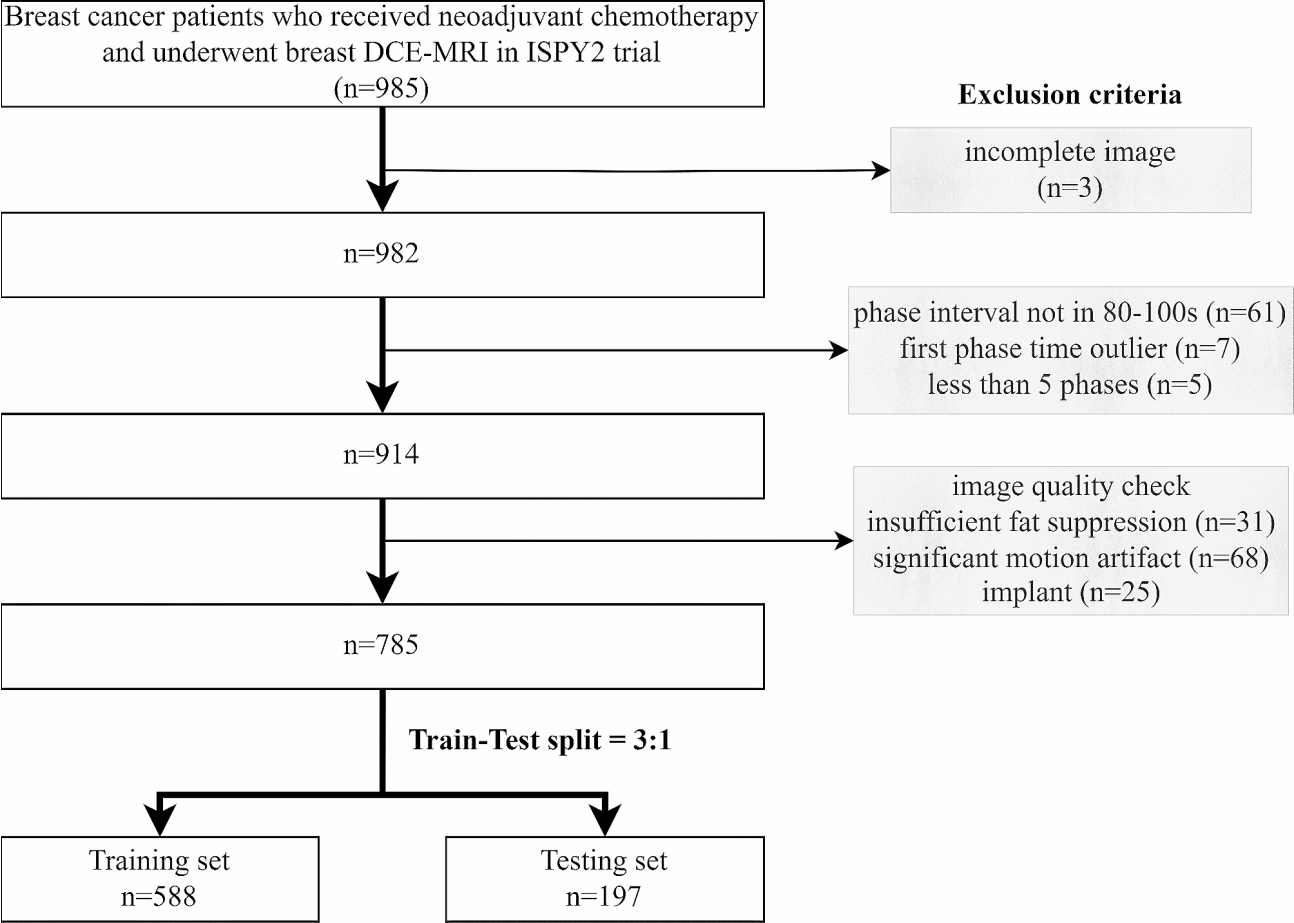
Patient cohort and train-test split

A total of 785 patients with complete imaging data and clinicopathological record constituted the entire patient cohort in this study and were divided into training set and testing set with a ratio of 3:1 (Fig. 2). As shown in Table 1, there is no significant difference in all of the patient characteristics between training set and testing set. The characteristics of pCR and non-pCR patients were tabulated in Supplementary Material Table S2. Significant association with pCR was observed in treatment, HR, HER2, and MP, while the other characteristics were independent of pCR.

**Table 1:** Patient characteristics in training set and testing set

		Train	Test	*P*-value
**Treatment**	Paclitaxel	95	47	0.448
	Paclitaxel + ABT 888 + Carboplatin	40	17	
	Paclitaxel + AMG 386	73	23	
	Paclitaxel + AMG 386 + Trastuzumab	12	4	
	Paclitaxel + Ganetespib	59	19	
	Paclitaxel + Ganitumab	69	20	
	Paclitaxel + MK-2206	32	14	
	Paclitaxel + MK-2206 + Trastuzumab	25	5	
	Paclitaxel + Neratinib	70	15	
	Paclitaxel + Pembrolizumab	44	10	
	Paclitaxel + Pertuzumab + Trastuzumab	23	10	
	Paclitaxel + Trastuzumab	16	4	
	T-DM1 + Pertuzumab	30	9	
**HR**	negative	265	89	1
	positive	323	108	
**HER2**	negative	454	157	0.322
	positive	144	40	
**MP**	negative	304	102	1
	positive	284	95	
**pCR**	negative	398	133	1
	positive	190	64	
**Age (mean, range)**		49 (25–77)	50 (23–72)	0.238
**Race**	American Indian or Alaska Native	4	0	0.434
	American Indian or Alaska Native; White	0	1	
	Asian	43	14	
	Asian; White	3	1	
	Black or African American	71	22	
	Native Hawaiian or Pacific Islander	4	0	
	Native Hawaiian or Pacific Islander; White	0	1	
	White	461	157	
	N/A	2	1	
**Menopausal status**	N/A	103	43	0.362
	Perimenopausal	21	7	
	Postmenopausal	177	64	
	Premenopausal	287	83	
**Ethnicity**	Hispanic or Latino	76	28	0.723
	Not Hispanic or Latino	511	169	
	N/A	1	0	

### Feature repeatability and feature change

The summarized results of feature repeatability and feature variation are shown in Supplementary Material Figure S2 and Figure S3. After removing the duplicate shape features and features with low repeatability, there were 480 radiomic features retained for each patient. For dynamic feature extraction, features repeatable in all the DCE-MRI phases and changing across different phases were retained. A total of 1232 dynamic features were extracted from 56 selected radiomic feature series for each patient and used in further analysis. An example of the dynamic feature is shown in Supplementary Material Table S3.

### Different models in pCR prediction

**Fig. 3:**
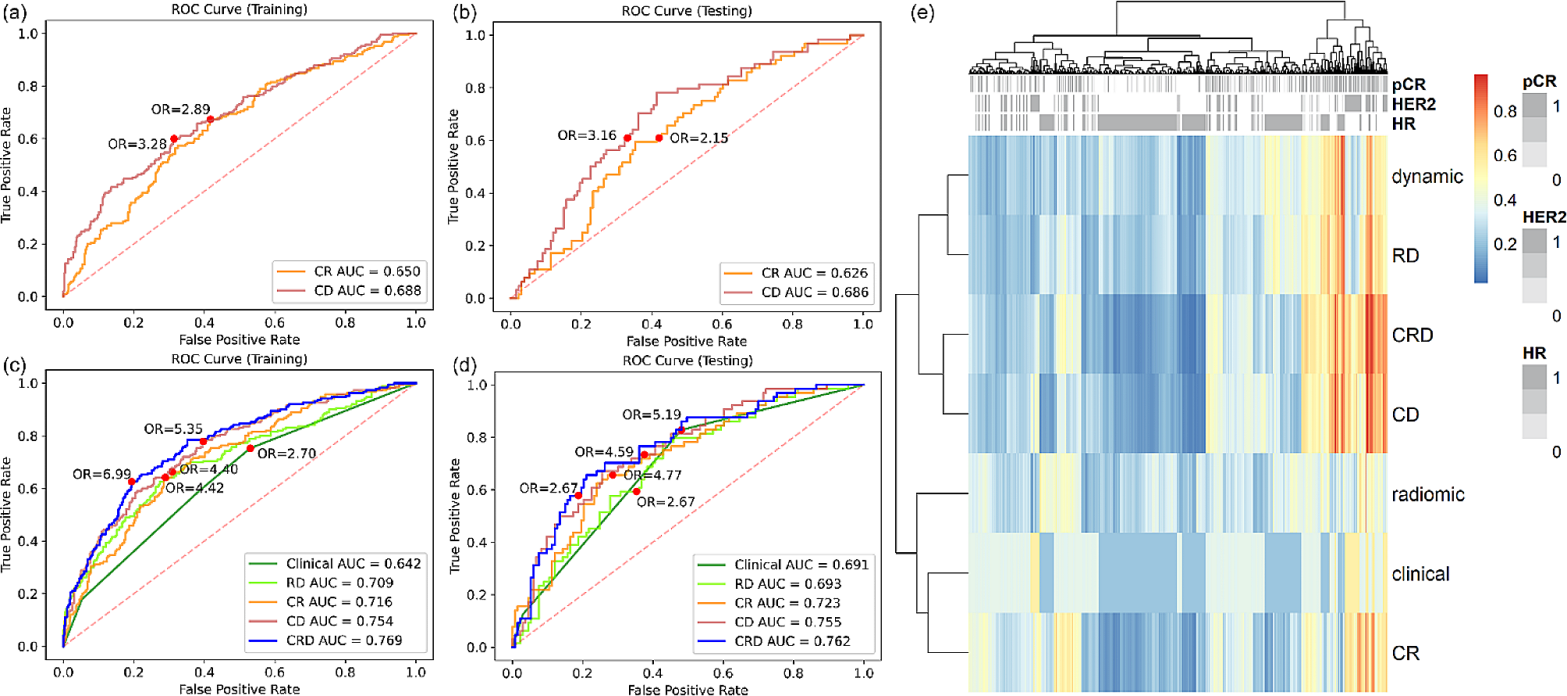
Reciever operating characteristic (ROC) curve analysis of dynamic model and radiomic model in (**a**) training set and (**b**) testing set. ROC analysis of clinical model, radiomic-dynamic (RD) model, clinical-radiomic (CR) model, clinical-dynamic (CD) model, and clinical-radiomic-dynamic (CRD) model in (**c**) training set and (**d**) testing set. (**e**) Heatmap of predicted probability by different models

A 10-feature dynamic model and a 4-feature radiomic model were developed separately (Supplementary Material Table S4). Dynamic model achieved higher AUC than radiomic model in both training set (0.688 vs. 0.650) and testing set (0.686 vs. 0.626) (Fig. 3(a)(b)), but the difference was not statistically significant (p value = 0.181 and 0.222). Dynamic model also had better performance in terms of accuracy and specificity, while the sensitivity was the same as radiomic model (Table 2). The significance of each feature in dynamic model and radiomic model was evaluated by the odds ratio and tabulated in Supplementary Material Table S5 and S6.

Among the clinicopathological variables provided in the dataset, treatment, HR, HER2, and MP were significantly associated with pCR (Supplementary Material Table S2). Since we intended to study biomarkers and MP requires expensive genomic test, only HR and HER2 were retained for further analysis. Table 3 summarized the metrics for pCR prediction performance of clinical model and combined models. Clinical-radiomic-dynamic (CRD) model achieved the highest training and testing AUC (Fig. 3(c)(d)), accuracy, and specificity, while clinical model had the highest sensitivity among all the models. The clinical factors, the dynamic model, and the radiomic model demonstrated independent value in CRD model as indicated by their coefficients and p values (Supplementary Material Table S7). Compared with clinical-radiomic (CR) model, CRD model shown significant improvement in both training and testing performance, indicating the additive value of dynamic model. Figure 3(e) shows the heatmap of the predicted probability by different models. Models containing dynamic features were clustered as similar models, showing the distinctive characteristic of dynamic features. The dynamic model was not associated with HR and HER2, demonstrating the independent value of dynamic model.

**Table 2:** Results of radiomic model and dynamic model for pCR prediction

	Training AUC	*p* value	Testing AUC	*p* value	Accuracy	Sensitivity	Specificity
**Dynamic model** ^#^	0.688 (0.635–0.741)		0.686 (0.594–0.778)		0.650	0.609	0.669
**Radiomic model**	0.650 (0.595–0.705)	0.181	0.626 (0.529–0.722)	0.222	0.589	0.609	0.579

P values were obtained by DeLong test

^#^ indicate reference model for comparison

Accuracy, sensitivity, and specificity were obtained in testing set

**Table 3:** Results of clinical model and combined models for pCR prediction

	Training AUC	*p* value	Testing AUC	*p* value	Accuracy	Sensitivity	Specificity
**CRD model** ^#^	0.769 (0.722–0.816)		0.762 (0.679–0.845)		0.736	0.578	0.812
**CD model**	0.754 (0.705–0.802)	< 0.001^*^	0.755 (0.672–0.839)	0.112	0.660	0.734	0.624
**CR model**	0.716 (0.665–0.767)	< 0.001^*^	0.695 (0.656–0.714)	0.005^*^	0.695	0.656	0.714
**RD model**	0.709 (0.658–0.761)	< 0.001*	0.693 (0.602–0.784)	< 0.001*	0.629	0.594	0.647
**Clinical model**	0.642 (0.586–0.697)	< 0.001^*^	0.691 (0.600-0.782)	< 0.001^*^	0.619	0.828	0.519

P values were obtained by likelihood ratio test

* indicate statistically significant ^#^ indicate reference model for comparison

Accuracy, sensitivity, and specificity were obtained in testing set

CRD model: Clinical-Radiomic-Dynamic model; CD model: Clinical-Dynamic model; CR model: Clinical-Radiomic model; RD model: Radiomic-Dynamic model

### Evaluation of the optimal model

**Fig. 4:**
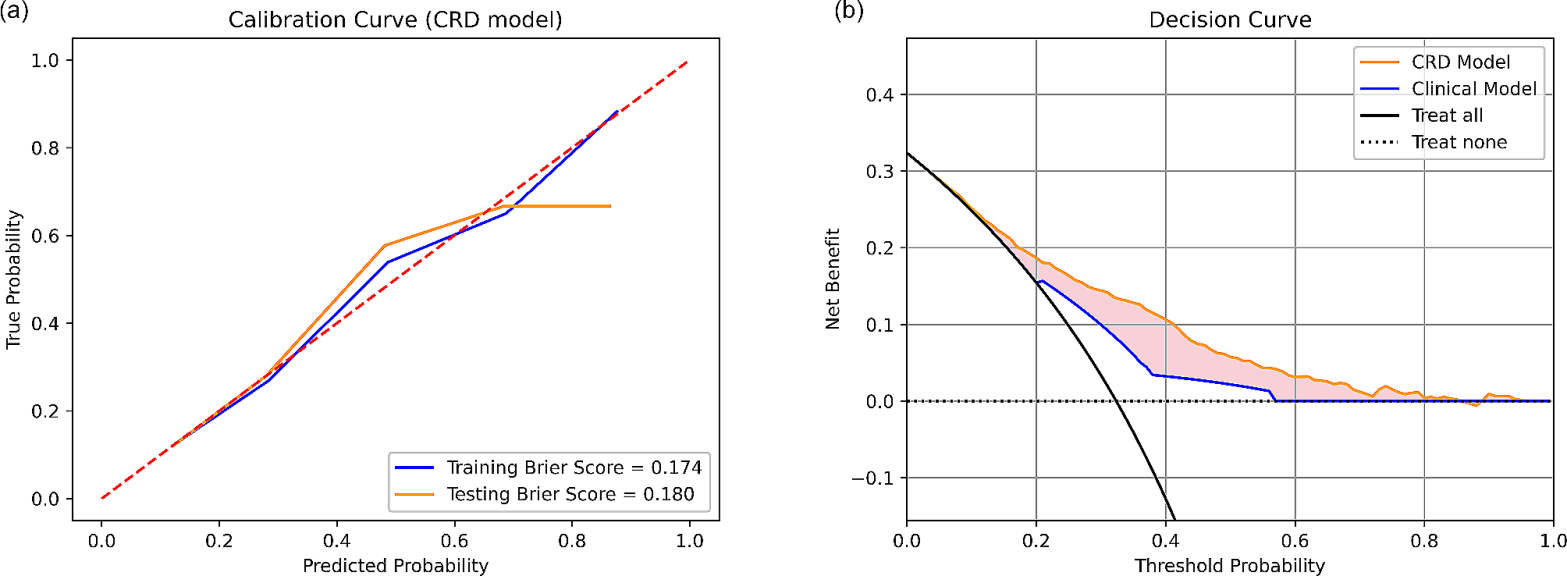
Calibration curve and decision curve analysis

Overall, CRD model has the best performance in pCR prediction. The calibration curves of CRD model had Brier score of 0.174 and 0.180 in training and testing set respectively (Fig. 4(a)), indicating well-alignment between predicted probablities and actual probabilities. The decision curve analysis of CRD model demonstrated its clinical usefulness by higher net benefit gain compared to clinical model and the other combined models (Fig. 4(b)).

**Fig. 5:**
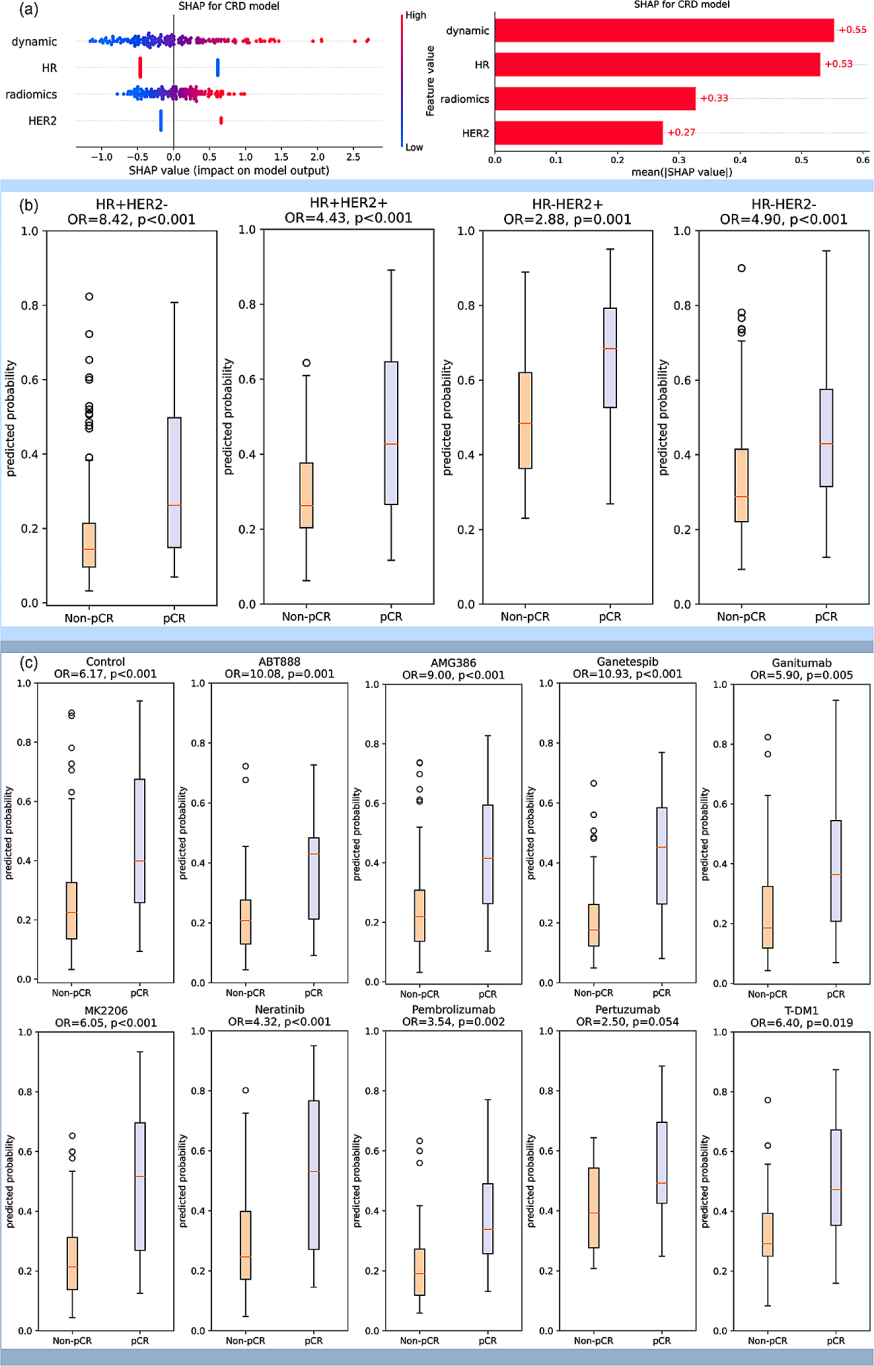
(**a**) SHAP analysis for interpretable component importance of CRD model. The beaswarm plot shows how each variable influence model output on single data where one dot represents one patient (left). The mean absolute SHAP value reflects the global effect of each variable on model output (right). (**b**) Box plots showing the predictive ability of CRD model in patient subgroups of various molecular subtypes. The molecular subtypes were defined by the status of HR and HER2, namely HR + HER2-, HR + HER2+, HR-HER2+, and HR-HER2-. The box plots indicate that the CRD model yields significantly distinct prediction probabilities for patients with pCR and non-pCR in all the four molecular subtypes. (**c**) Box plots showing the predictive ability of CRD model in patients receiving various treatments. Patients received standard care (control) or standard care plus one trial agent (ABT888, AMG386, Ganetespib, Ganitumab, MK2206, Neratinib, Pembrolizumab, Pertuzumab, T-DM1) in the trial. The box plots suggest that CRD model demonstrates the capability to differentiate pCR and non-pCR patients across various treatment drugs in this trial, with the exception of a marginal significance observed in Pertuzumab. All the p values were obtained by student t test. OR: odds ratio

The SHAP value analysis of CRD model shown high importance of dynamic model, which was comparable to HR. The importance of radiomic model and HER2 was a little bit lower, but still had significant effect on the model output (Fig. 5(a)). The CRD model was also used in stratifying pCR and non-pCR patients under different pre-defined molecular subtypes. It shown a significant stratification ability in all the four molecular subtypes with odds ratio (OR) of 2.88–8.42. (Fig. 5(b)). In the analysis of patients receiving different drugs, except for the marginally significant performance in Pertuzumab arm, CRD model shown significant association with pCR with OR of 2.88–10.93 in the other treatment arms (Fig. 5(c)).

### DEGs and enriched pathways

**Fig. 6:**
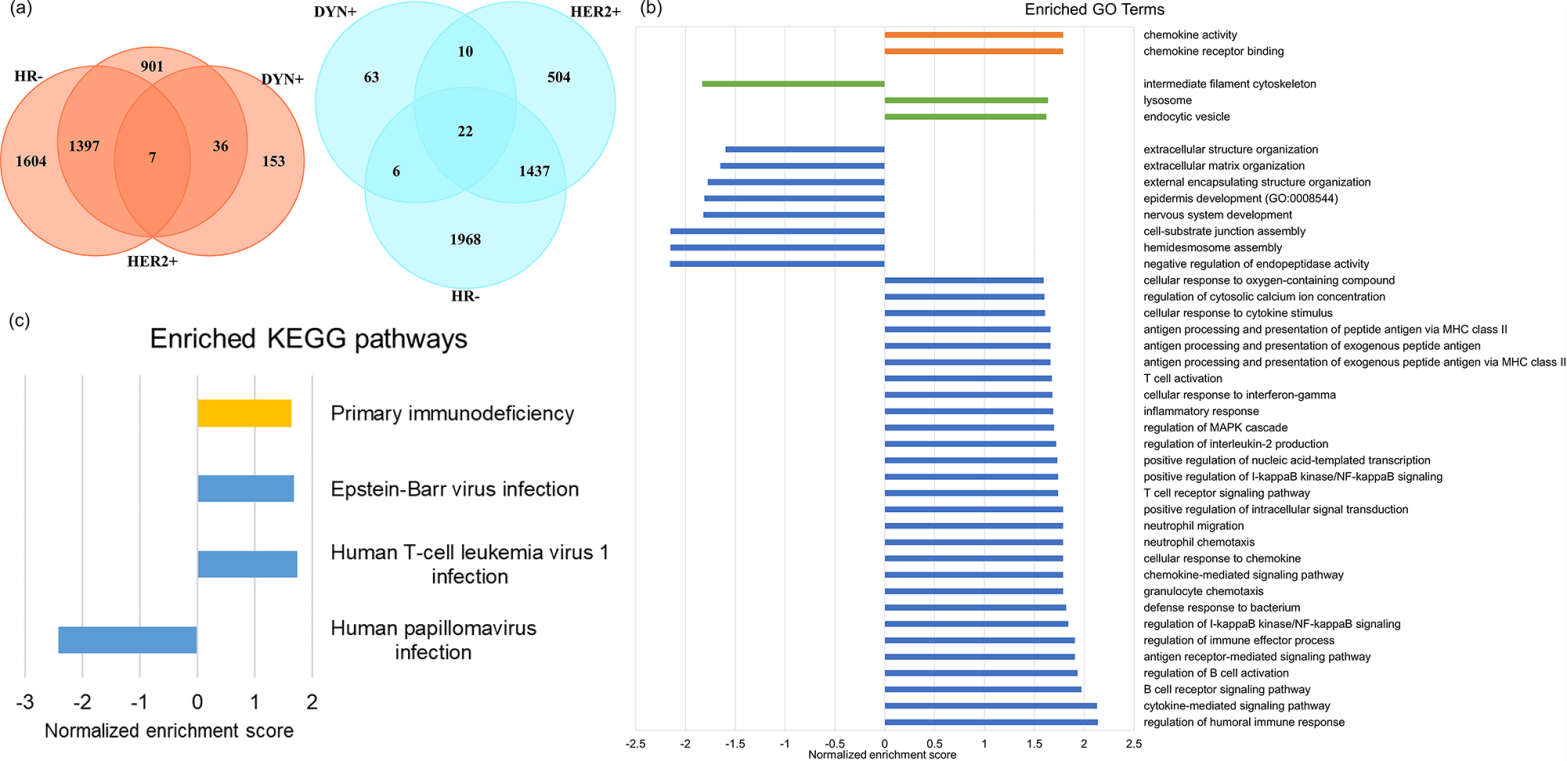
(**a**) Up-regulated (left) and down-regulated (right) DEGs in DYN+, HR-, and HER2 + group. (**b**) Enriched GO terms in DYN + group. (**c**) Enriched KEGG pathways in DYN + group

In DEG analysis, a total of 196 up-regulated genes and 101 down-regulated genes in DYN + group were identified. As compared with HR- group and HER2 + group, which also associate with better pCR outcome in ISPY2 trial, there are 7 common up-regulated genes and 22 common down-regulated genes (Fig. 6(a)). In GSEA by GO terms, there are 36 biological processes, 3 cellular components, 2 molecular functions enriched in DYN+ (Fig. 6(b)), many of which are associated with immune system. There are 4 enriched pathways in GSEA by KEGG, in which 3 pathways are related to viral disease and 1 pathway is related to immune disease (Fig. 6(c)).

## Discussion

While the dynamic information in DCE-MRI has shown potential in various clinical applications, the exploration of DCE-MRI-derived radiomic feature series has remained limited. This study systematically extracted dynamic features from DCE-MRI-derived radiomic feature series using feature-based time series analysis method and built dynamic model for pCR prediction. Adding dynamic model to exisitng clinical and radiomic model can improve pCR prediction. Radiogenomic analysis revealed correlations of dynamic model with some breast cancer prognosis-related genes and pathways, providing the potential biological explanations for the additive value.

The change in DCE-MR image appearances caused by the flow of contrast agent may contain valuable information for pCR prediction. Previous studies have employed delta features and statistical distributions to characterize the relevant dynamic information [12, 13]. However, the former method may provide limited information by utilizing only two of multiple DCE-MR phases, while the latter method disregards the temporal information that is crucial for reflecting the directional flow of contrast agent. A recently published paper implemented several classical time series analysis algorithms in DCE-MRI-derived radiomic feature series and achieved an accuracy of 0.852 in breast cancer diagnosis, demonstrating the significance of serial information as well as the feasibility and efficacy of time series analysis [46]. In our study, we used radiomic features to comprehensively describe DCE-MR image appearance and adopted Catch22 to systematically analyze the dynamics of radiomic feature series. The Catch22 feature set takes into account both the temporal order and relative magnitude of series values. It has been successfully implemented in many time series analysis applications, such as breath signal and heart rate. To the best of our knowledge, it is the first study to apply a systematic feature-based time series analysis method to DCE-MRI for pCR prediction. Our results demonstrated the utility of the extracted dynamic features by showing a modestly higher AUC of dynamic model in comparison to the conventional radiomic model. Furthermore, the dynamic features provided additive value to the existing methods, as evidenced by a significantly improved model performance compared with both clinical model and CR model. Overall, we have demonstrated the feasibility and efficacy of extracting dynamic information through feature-based time series analysis and the potential of dynamic features in facilitating pCR prediction. Besides, our method offers the advantage of interpretability as Catch22 provides clear definition for each dynamic feature. And it is adaptable to different time series length which is frequently encountered in real-world clinical practice due to the variations of machines and scan settings. Our method demonstrates the potential to be implemented in real clinical practice, although further validation is required to confirm its performance in diverse settings.

Both single-modal and multi-modal models were developed in this study. While the imaging-based model and clinical model appeared to have similar performance, the combined models shown better performance than individual models. The CRD model achieved the highest AUC, which is significantly better than RD model and clinical model alone, indicating that imaging features and clinical factors may provide distinct and complementary information for pCR prediction. Subgroup analysis of the CRD model was conducted to further explore the effectiveness of CRD model under various conditions. Breast cancer is a highly heterogeneous disease characterized by various HR and HER2 status, resulting in four molecular subtypes. Our results on molecular subtype analysis resulted in varying effect size by OR ranging from 2.88 to 8.42, where a larger OR indicates a stronger predictive ability. While CRD model is significantly associated with pCR in all the molecular subtypes, our results suggested that CRD model has stronger predictive ability for patients of HR + HER2-. The CRD model was also evaluated by its effect for patients receiving different drugs, resulting in the largest OR in Ganetespib and marginally significant OR in Pertuzumab. This indicates the various predictive value of CRD model for various treatment drugs and assists the clinicians to decide applicable scenarios. In general, CRD model shown generalizability acorss various molecular subtypes and various treatment drugs. However, due to the nature of trial data, the patient numbers are small in each subgroups and further validation on larger cohort is required to confirm the results.

It is believed that radiomics is able to detect the underlying biological processes in the human body by analyzing image textures that are imperceptible to human eyes. Moreover, pre-treatment radiomics mostly reflect the baseline tumor characteristics, which is the result of various biological processes and associated with treatment response. Previous studies indicated the representativeness of image phenotypes for the biological characteristics by demonstrating their similar predictive ability to pCR [47]. However, few radiomics study has linked the image phenotype to biological processes through radiogenomic analysis [48]. In this study, we conducted a radiogenomic analysis to associate our dynamic model to the genomic profiles of breast tumors, providing insight into the underlying biological mechanisms of radiomics. Some DEGs in our DYN + subgroup is associated with better prognosis of breast cancer patients. For example, the DYN + subgroup has higher expression of CXCL9 which was demonstrated to associate with higher pCR rate in breast cancer patients receiving NAC in previous study [49]; HLA-DPB1 was up-regulated in DYN + subgroup and was also assocaited with more tumor infiltrating lymphcytes and thereby better prognosis [50]. On the other hand, DEGs such as CCL18 is associated with angiogenesis in breast cancer, demonstrating the potential of DYN to represent the dynamics in DCE-MRI [51].

Our study has several limitations. Due to the retrospective nature of the study, it is possible that our results suffer from spectrum bias and information bias, which may compromise the overall strength of evidence of our study. Besides, our study included a medium sample size (*n* = 785) without external validation, which may not be representative enough for the large population of breast cancer patients. Therefore, further study is needed to externally validate our methods and conclusions in a prospective manner. Also, our study only employed DCE-MRI, while multi-parametric MR images could be available in clinics. Further exploration on incorporating other MR images is warranted.

## Conclusions

In conclusion, this study quantified the dynamic characteristics of DCE-MRI by calculating dynamic properties of radiomic feature series and developed a dynamic model. The dynamic model can aid in improving pCR prediction of breast cancer patients receiving NAC. The potential biological underpinnings of the dynamic model was explored by demonstrating its association with tumor heterogeneity in gene expression. Further investigations on more biological associations and assisting treatment selection are warranted.

### Electronic supplementary material

Below is the link to the electronic supplementary material.

Supplementary Material 1

## Data Availability

The dataset used in this study is a public dataset available at The Cancer Image Archive with accession code 70230072. Dynamic and radiomics features that support the findings of this study can be found here: https://github.com/Xinyu-Z000/DCE-MRI-dynamics.
