# Supplementary material for: Enhancing pathological complete response prediction in breast cancer: the role of dynamic characterization of DCE-MRI and its association with tumor heterogeneity
Source: Breast Cancer Res. 2024 May 14;26:77. doi: 10.1186/s13058-024-01836-3 (PMC11094888; doi:10.1186/s13058-024-01836-3)
Supplement: Supplementary file 1 — Supplementary Material 1 [file 13058_2024_1836_MOESM1_ESM.docx]

Figure S1 – Image preprocessing

Before feature extraction, several steps were involved to preprocess images: (1) use threshold to exclude voxels that didn’t have an intensity within the range of (mean-standard deviation, mean+8*standard deviation), to remove the noise; (2) normalize images using z-score normalization with z=100; (3) resample images to 1*1*1 mm^3^; (4) discretize images by a fixed bin width of 5. Below image shows the distribution of intensity range of each patient after completing image preprocessing. The intensity range had a mean of 710 and standard deviation of 203.


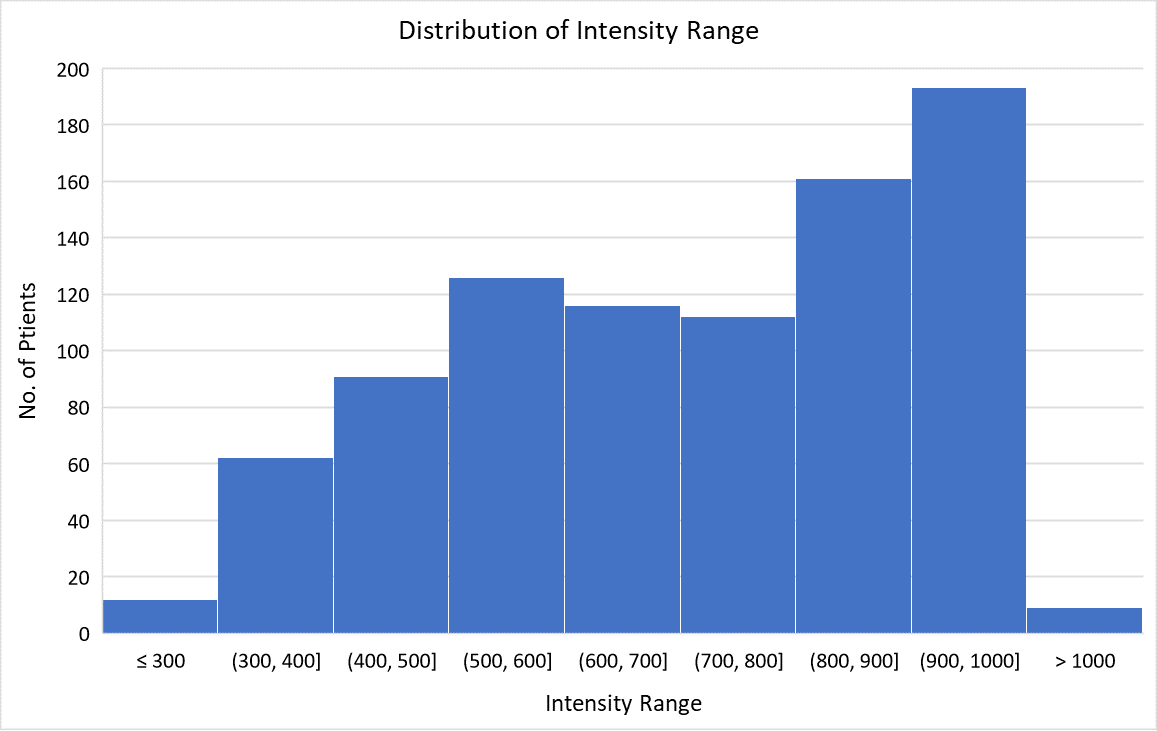


Figure S2. Feature repeatability of different DCE-MRI phases.

The repeatability of extracted radiomics features was assessed by single rater intraclass correlation coefficient (ICC) (1,1). The ICC for each feature was calculated under 30-time perturbations which involved random translation, rotation, as well as contour randomization. The formula for ICC calculation is:

$$\frac{MS_{R}-MS_{W}}{MS_{R}+\left( k+1 \right)MS_{W}}$$

where MS_R_ is the mean square for all the features, MS_W_ is the mean square for the residual sources of variance, and k is the number of measurements. The calculation of ICC was performed using python package pingouin (version 0.5.2). An ICC >= 0.9 was determined as high-repeatable. Below figures shown the feature repeatability at different DCE-MRI phases.


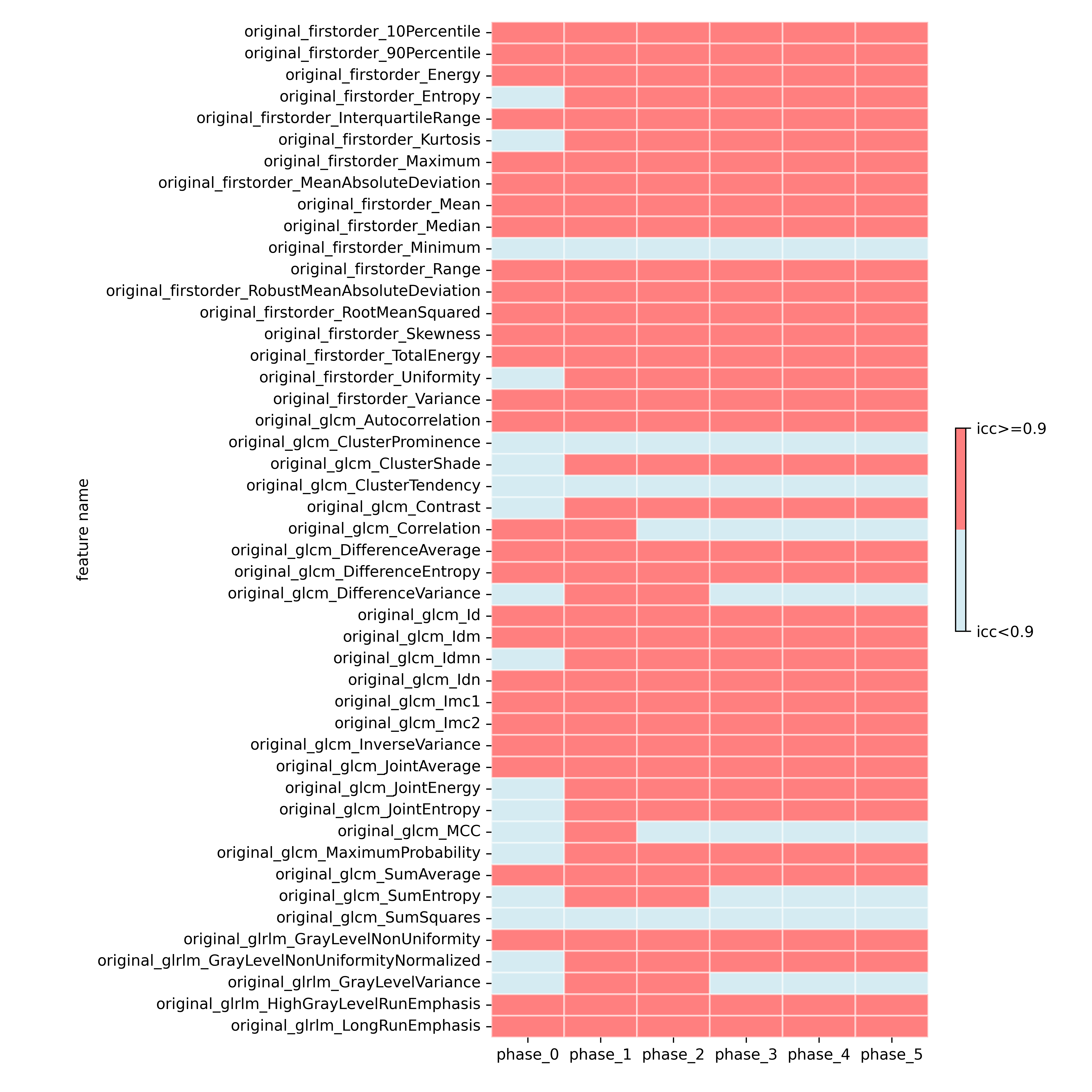

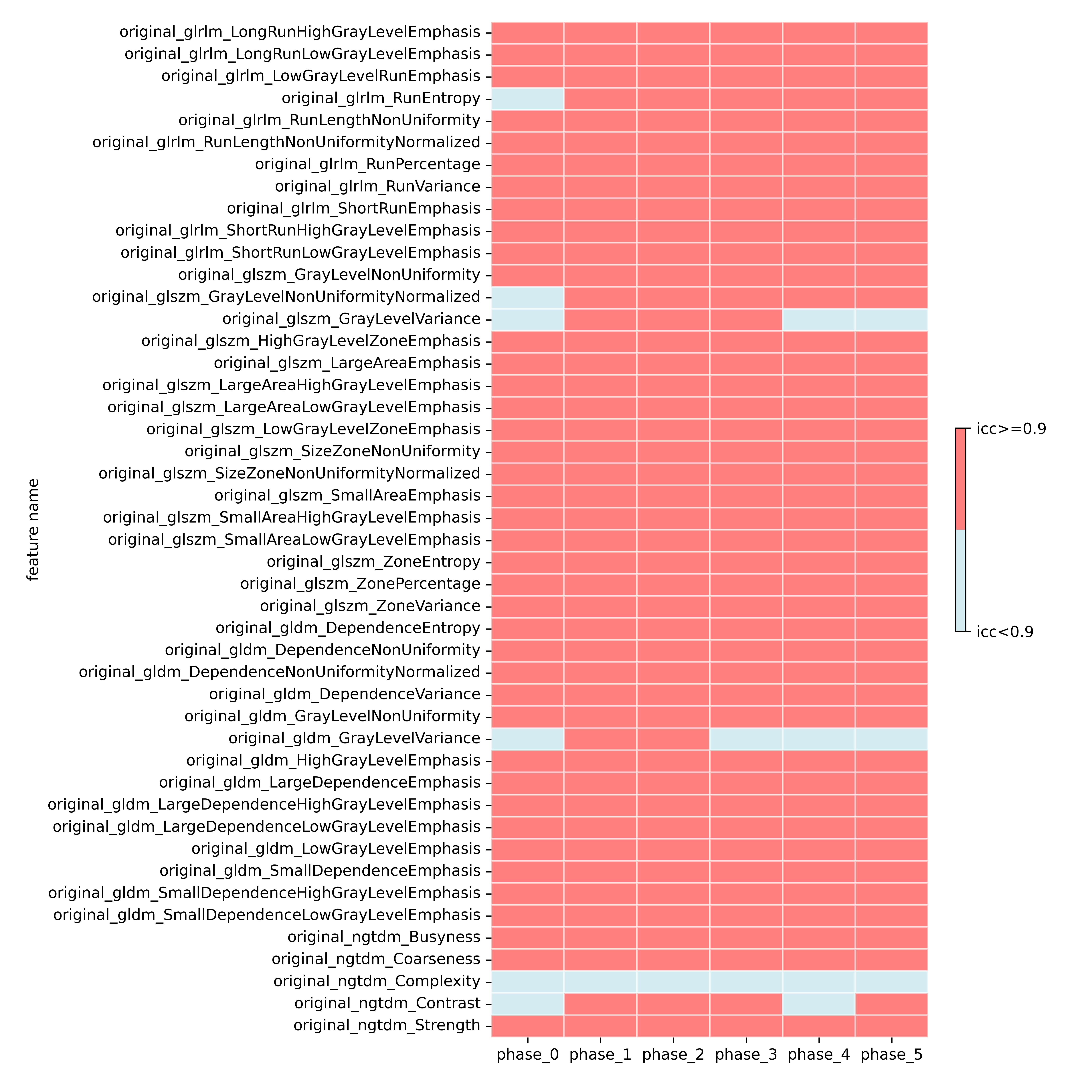


Figure S3. Feature change across different DCE-MRI phases.

The assessment of feature change across different DCE-MRI phases involved evaluating whether the feature change calculated by subtraction was significantly different from zero. It was conducted by performiang a single-sample t test. A t test p value smaller than 0.05 was determined as ‘change’. Below figures shown the feature change across different phases. The coordinate of x-axis n/m referred to feature change = feature at phase n – feature at phase m, and 0-5 denoted pre-contrast phase and 1^st^ to 5^th^ post-contrast phase.


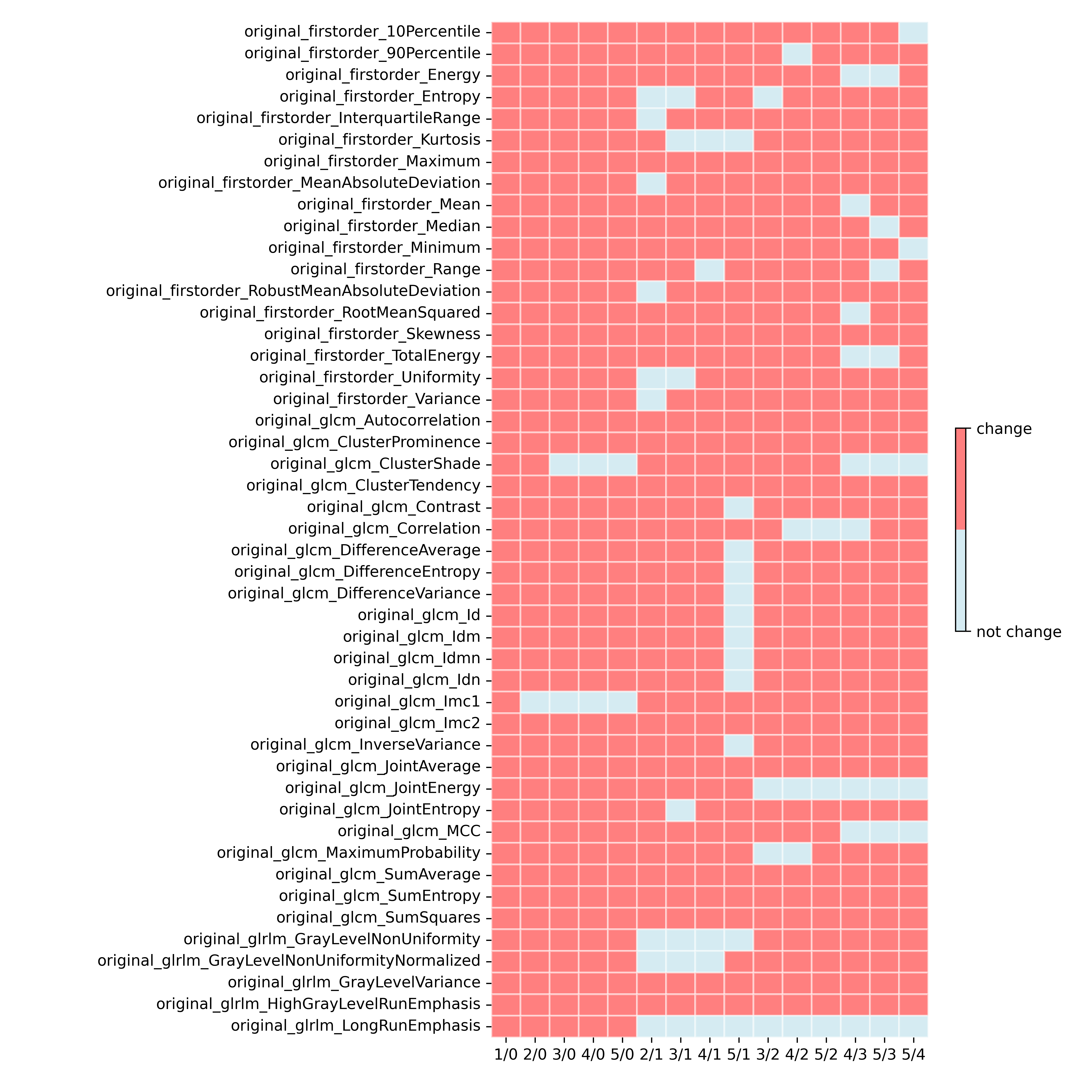

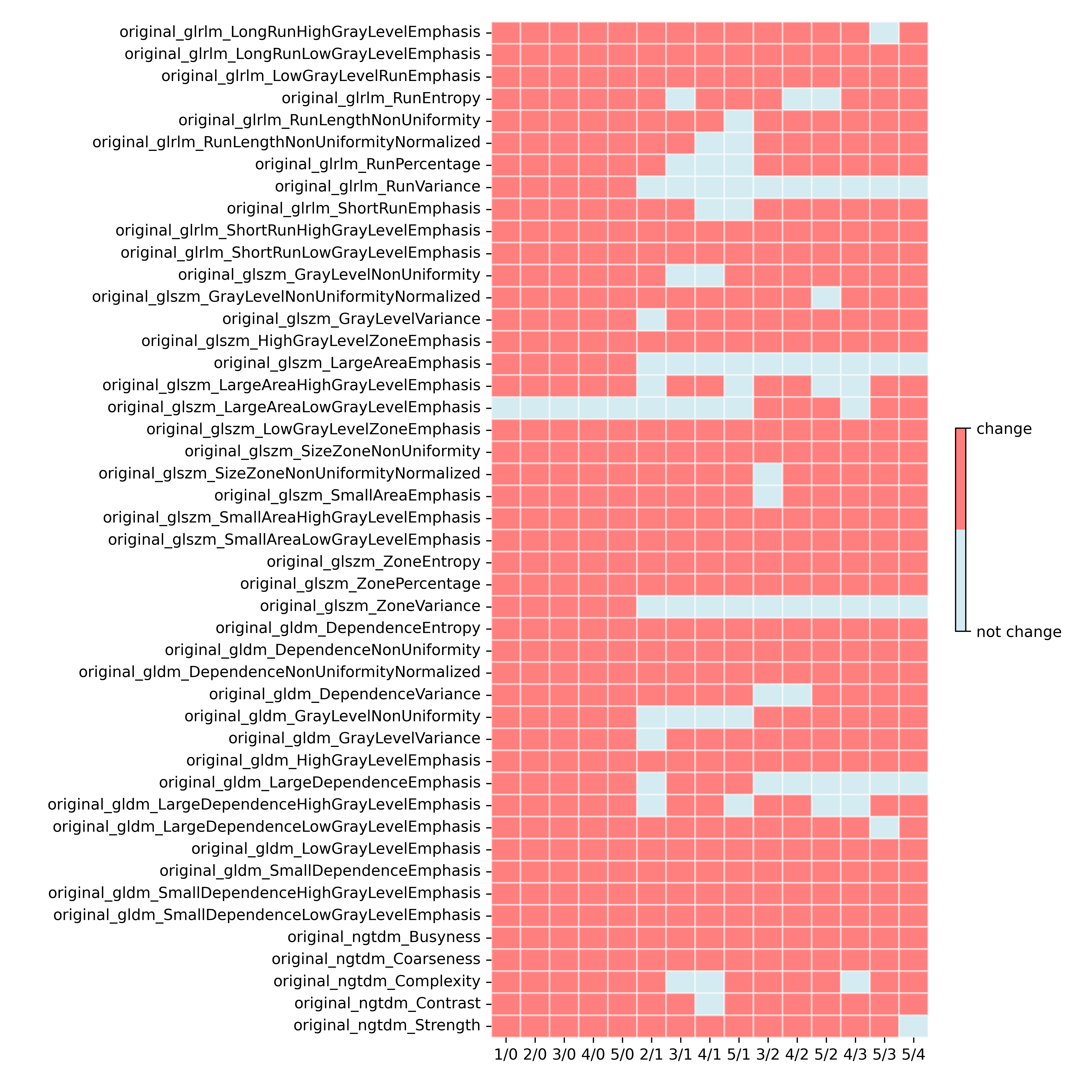


Table S1. DCE-MRI parameters

| **Parameter** | **Value** |
| --- | --- |
| Scanner Manufacturer | GE / Philips / SIEMENS |
| Magnetic Field Strength | 1.5T / 3.0T |
| Fat Suppression | Required |
| Slice Orientation | Axial |
| Slice Thickness | 0.8 - 3 mm |
| Pixel Size | 0.3 - 1.4 mm |
| Slice Number | 56 - 256 |
| Repetition Time | 3.8 - 9.3 s |
| Echo Time | 1.3 - 4.8 s |
| Number of averages | 0.7 - 3 |
| Spacing Between Slices | 0.8 - 2.6 mm |
| Flip Angle | 10 - 20 degrees |
| Total Phase | 6 - 11 |
| Sequence Acquisition Time | 80 - 100 s |
| Total Acquisition Time | At least 8 min after contrast agent injection |

Table S2. Patient characteristics of pCR and non-pCR.

|  |  | **non-pCR** | **pCR** | **P-value** |
| --- | --- | --- | --- | --- |
| **Treatment** | Paclitaxel | 114 | 28 | <0.001 |
|  | Paclitaxel + ABT 888 + Carboplatin | 36 | 21 |  |
|  | Paclitaxel + AMG 386 | 67 | 29 |  |
|  | Paclitaxel + AMG 386 + Trastuzumab | 10 | 6 |  |
|  | Paclitaxel + Ganetespib | 58 | 20 |  |
|  | Paclitaxel + Ganitumab | 69 | 20 |  |
|  | Paclitaxel + MK-2206 | 32 | 14 |  |
|  | Paclitaxel + MK-2206 + Trastuzumab | 15 | 15 |  |
|  | Paclitaxel + Neratinib | 54 | 31 |  |
|  | Paclitaxel + Pembrolizumab | 29 | 25 |  |
|  | Paclitaxel + Pertuzumab + Trastuzumab | 13 | 20 |  |
|  | Paclitaxel + Trastuzumab | 17 | 3 |  |
|  | T-DM1 + Pertuzumab | 17 | 22 |  |
| **HR** | negative | 201 | 153 | <0.001 |
|  | positive | 330 | 101 |  |
| **HER2** | negative | 432 | 169 | <0.001 |
|  | positive | 99 | 85 |  |
| **MP** | negative | 312 | 94 | <0.001 |
|  | positive | 219 | 160 |  |
| **Age(mean,range)** |  | 49 (23-77) | 49 (25-73) | 0.530 |
| **Race** | American Indian or Alaska Native | 3 | 1 | 0.791 |
|  | American Indian or Alaska Native;White | 0 | 1 |  |
|  | Asian | 39 | 18 |  |
|  | Asian;White | 3 | 1 |  |
|  | Black or African American | 63 | 30 |  |
|  | Native Hawaiian or Pacific Islander | 2 | 2 |  |
|  | Native Hawaiian or Pacific Islander;White | 0 | 1 |  |
|  | White | 419 | 199 |  |
|  | N/A | 2 | 1 |  |
| **Menopausal status** | Premenopausal | 259 | 111 | 0.291 |
|  | Perimenopausal | 18 | 10 |  |
|  | Postmenopausal | 152 | 89 |  |
|  | N/A | 102 | 44 |  |
| **Ethnicity** | Hispanic or Latino | 66 | 38 | 0.573 |
|  | Not Hispanic or Latino | 464 | 216 |  |
|  | N/A | 1 | 0 |  |

Table S3 – Example of visualizing dynamic features

The table gives an example of how a dynamic feature quantify the change of a radiomics feature. The selected radiomics feature is original_glszm_LowGrayLevelZone_Emphasis. The dynamic feature is MD_hrv_classic_pnn40. It calculates the proportion of differences magnitude that is greater than 4% of standard deviation of the time series. In these figures, the x-axis denotes the phase of DCE-MRI and y-axis denotes the value of radiomics feature. And each line denotes one patient. Those have a relatively constant radiomics feature value after contrast agent injection (phase1 to phase5) were given lower dynamic feature value. This table also shows that patients with different dynamic feature value has d0i00fferent pCR rate.

|  | 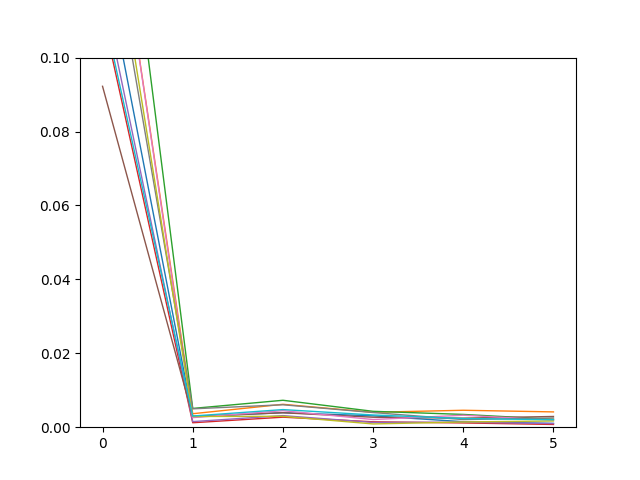 | 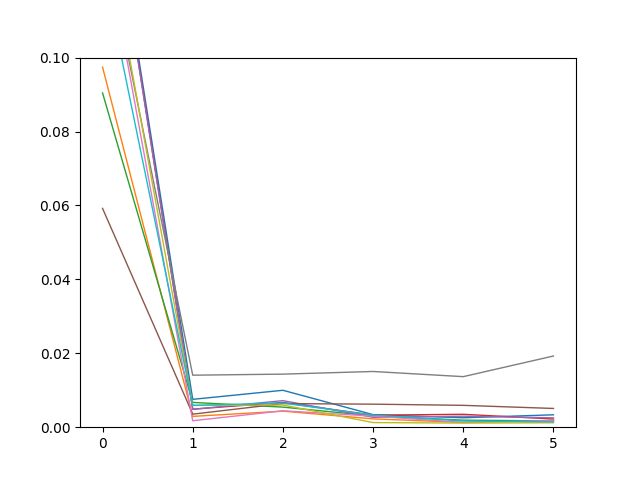 |
| --- | --- | --- |
| Feature value | -3.53 | -1.91 |
| pCR rate  (pCR / total) | 0.2 (2/10) | 0.25 (8/32) |
|  | 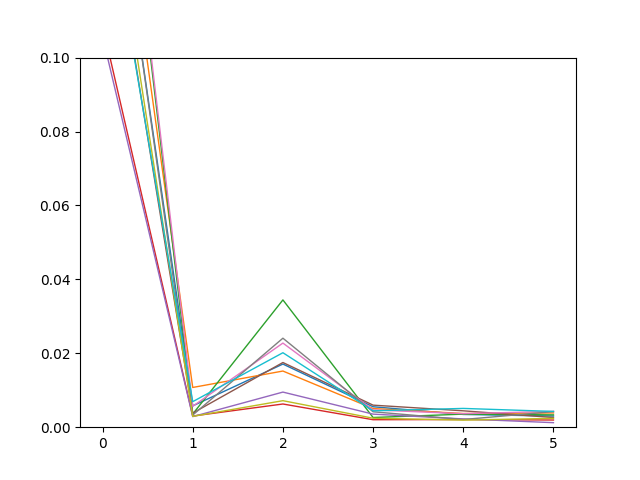 | 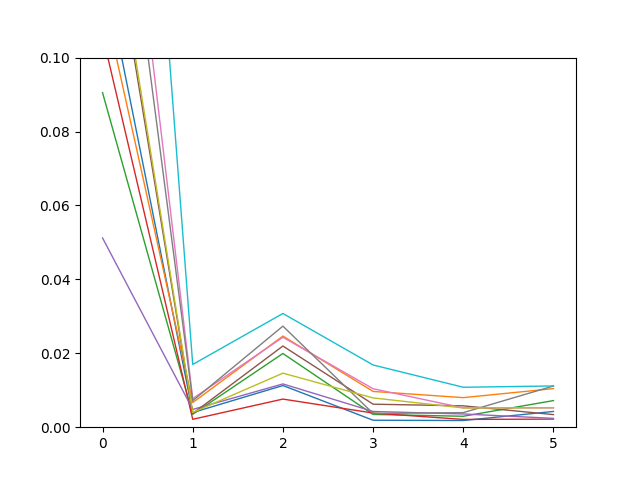 |
| Feature value | -0.29 | 1.33 |
| pCR rate  (pCR / total) | 0.32 (185/582) | 0.38 (50/133) |
|  | 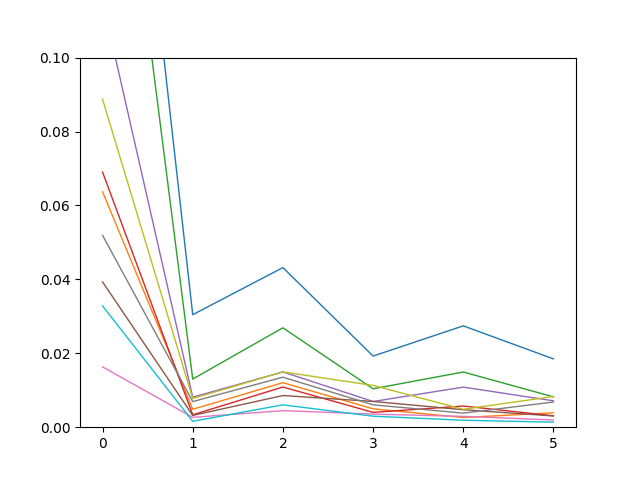 |  |
| Feature value | 2.95 |  |
| pCR rate  (pCR / total) | 0.30 (9/30) |  |

Table S4 Performance of models with different feature number.

| Dynamic model feature No. | Training AUC | Internal Validation AUC | Radiomic model feature No. | Training AUC | Internal Validation AUC |
| --- | --- | --- | --- | --- | --- |
| 1 | 0.571 | 0.560 | 1 | 0.595 | 0.564 |
| 2 | 0.600 | 0.634 | 2 | 0.621 | 0.535 |
| 3 | 0.607 | 0.635 | 3 | 0.650 | 0.637 |
| 4 | 0.643 | 0.649 | **4** | **0.650** | **0.640** |
| 5 | 0.650 | 0.675 | 5 | 0.650 | 0.634 |
| 6 | 0.660 | 0.643 | 6 | 0.650 | 0.635 |
| 7 | 0.664 | 0.673 | 7 | 0.666 | 0.603 |
| 8 | 0.675 | 0.667 | 8 | 0.672 | 0.603 |
| 9 | 0.682 | 0.660 | 9 | 0.673 | 0.589 |
| **10** | **0.688** | **0.680** | 10 | 0.690 | 0.570 |
| 11* | 0.698 | 0.651 |  |  |  |

*As the 10-feature dynamic model achieved the highest internal validation AUC. We further explored whether adding more dynamic features can obtain better performance. Therefore, the results of 11-feature dynamic model is also listed here.

Table S5 Odds ratios showing the significance of dynamic features in the dynamic model.

| Dynamic feature name | Odds ratio |
| --- | --- |
| original_firstorder_RootMeanSquared_SP_Summaries_welch_rect_centroid | 1.098 |
| original_firstorder_Mean_FC_LocalSimple_mean3_stderr | 0.795 |
| original_glrlm_ShortRunLowGrayLevelEmphasis_SP_Summaries_welch_rect_centroid | 0.807 |
| original_glszm_GrayLevelNonUniformity_CO_HistogramAMI_even_2_5 | 0.780 |
| original_glszm_LowGrayLevelZoneEmphasis_MD_hrv_classic_pnn40 | 1.161 |
| original_glcm_Id_SB_MotifThree_quantile_hh | 0.793 |
| original_glcm_DifferenceEntropy_CO_HistogramAMI_even_2_5 | 1.193 |
| original_firstorder_90Percentile_FC_LocalSimple_mean1_tauresrat | 1.248 |
| original_glcm_Imc1_CO_f1ecac | 0.868 |
| original_firstorder_InterquartileRange_DN_HistogramMode_10 | 0.833 |

Table S6 Odds ratios showing the significance of radiomic features in the radiomic model.

| Radiomic feature name | Odds ratio |
| --- | --- |
| original_shape_Maximum2DDiameterColumn_phase0 | 0.793 |
| original_glcm_Imc2_phase1 | 1.345 |
| original_glszm_ZoneEntropy_phase0 | 0.772 |
| original_ngtdm_Busyness_phase1 | 0.973 |

Table S7 Coefficients and p valus of variables in the CRD model

| Variable | coefficient | P value |
| --- | --- | --- |
| HR | -1.076 | <0.001 |
| HER2 | 0.837 | <0.001 |
| Dynamic | 4.879 | <0.001 |
| Radiomic | 4.169 | <0.001 |
